# Errors in the Spontaneous Language of Survivors of Pediatric Cerebellar Tumors

**DOI:** 10.1007/s12311-024-01754-2

**Published:** 2025-01-07

**Authors:** Cheyenne Svaldi, Juan-Ignacio Galli, Philippe Paquier, Stefanie Keulen, Henrieke Van Elp, Coriene Catsman-Berrevoets, Annet Kingma, Roel Jonkers, Saskia Kohnen, Vânia de Aguiar

**Affiliations:** 1 Center for Language and Cognition, University of Groningen, PO box 716, 9700 AS Groningen, the Netherlands; 2 Brussels Centre for Language Studies (BCLS), Language, Brain and Cognition, Vrije Universiteit Brussel (VUB), Pleinlaan 2, 1050 Brussels, Belgium; 3 Centre for Research in Cognition and Neurosciences (CRCN), Université Libre de Bruxelles (ULB), Avenue Franklin D. Roosevelt 50, 1050 Brussels, Belgium; 4 Department of Translational Neurosciences (TNW), Universiteit Antwerpen (UA), Universiteitsplein 1, 2610 Antwerp, Belgium; 5 Department of Paediatric Neurology Erasmus Medical Centre, Sophia Children’s Hospital Rotterdam, Doctor Molewaterplein 40, 3015 GD Rotterdam, the Netherlands; 6 Department of Paediatrics, University Medical Centre Groningen, Hanzeplein 1, 9700 RB Groningen, the Netherlands; 7 Australian Centre for the Advancement of Literacy, Australian Catholic University, Berrystreet 23, North Sydney, New South Wales, 2060 Australia; 8 Department of Radiation Oncology, University Medical Center Groningen, Groningen, the Netherlands

**Keywords:** Cerebellum, Postoperative cerebellar mutism syndrome, Spontaneous language, Posterior fossa surgery

## Abstract

Pediatric cerebellar tumor survivors may present with spontaneous language impairments following treatment, but the nature of these impairments is still largely unclear. A recent study by Svaldi et al. (Cerebellum. 23:523-44, 2023) found a broad spectrum of spontaneous language impairments irrespective of postoperative cerebellar mutism syndrome (pCMS) diagnosis in long-term pediatric cerebellar tumor survivors. Several patients presented with reduced grammatical or lexical accuracy, but a detailed error analysis was lacking. The present study builds on this study by 1) investigating the error types in spontaneous language across three language processing levels in the same participant group and 2) by evaluating the possible association between pCMS and the processing nature of the language impairments. Spontaneous language was evaluated in 12 long-term survivors of pediatric cerebellar tumors (*M*(*SD*) = 4;8(3;8) years), of whom five were diagnosed with pCMS. The proportion of occurrence of each error type was compared between each patient and five matched controls using individual case statistics, reflecting (lexico-)phonological (i.e., phonemic paraphasias), lexical-semantic (e.g., empty speech) and morphosyntactic processing (e.g., verb inflection errors). Each patient showed a significantly higher proportion of at least one of the included error types across all language processing levels. A higher proportion of general-all-purpose verbs and inaccurate verb inflection were the most common errors and respectively reflected lexical-semantic and morphosyntactic processing. Additional language impairments were identified using the error analysis that were not identified with standard language measures and psycholinguistic analysis, suggesting the added diagnostic value of error analyses.

**Supplementary Information:**

The online version contains supplementary material available at 10.1007/s12311-024-01754-2.

## Introduction

Because of an increase in long-term survival in children with cerebellar tumors [1], interest has risen in identifying long-term sequelae of the tumor, the tumor treatment and the need for cognitive rehabilitation [2]. Apart from a broad range of motor and cognitive impairments, cerebellar tumor survivors may present with speech (e.g., dysarthria) and language impairments (e.g., agrammatism) [3–5]. Although 25% to 35% of children present with postoperative cerebellar mutism syndrome (pCMS), characterized by severe language deficits, this is not a prerequisite for language difficulties [4, 6].

Previous studies describe language disorders in pediatric cerebellar tumor survivors based on formal language measures (e.g., picture naming task; assessing a specific language function) [3, 5], but also in spontaneous language analyses (e.g., storytelling; assessing multiple language functions simultaneously) [5, 7, 8]. Nonetheless, comprehensive reports of strengths and weaknesses at specific levels of language processing are lacking in the literature though these are necessary for targeted language rehabilitation [2, 6]. Recently, Svaldi et al. [7] provided a detailed spontaneous language analysis across four language processing levels (i.e., semantic, lexical, phonological, morphosyntactic) using standard measures and psycholinguistic properties. Impairments were identified across all levels of language processing, irrespective of pCMS diagnosis. Importantly, several patients presented with reduced lexical and/or grammatical accuracy but an error analysis was lacking. Therefore, the present study will build on this work by 1) characterizing the types of (lexico-)phonological, lexical-semantic and morphosyntactic errors in the spontaneous language of pediatric cerebellar tumor survivors and 2) examining the potential association between pCMS and the nature of the language impairments. The participant group is the same as reported by Svaldi et al. [7].

## Methods

### Participants

The patient group consisted of 12 (five females, seven males) long-term survivors of pediatric cerebellar tumors with a mean age at assessment of 11;3 years (*SD* = 6;3 years, range = 3–24;2 years). Patients were recruited via the Erasmus Medical Centre/Sophia Children´s Hospital in Rotterdam as part of an earlier study [9] and time since surgery ranged between 11 months and 12;3 years (*M*(*SD*) = 4;8(3;8) years). Five patients suffered from pCMS. Five cerebellar tumor survivors were diagnosed with a medulloblastoma, five with a pilocytic astrocytoma, and two with an ependymoma.

Control data were gathered until each patient could be matched in age and gender with five controls resulting in a total of 39 participants with no history of language or learning impairment (*M*(*SD*) = 11;1(5;11) years; range = 3;0–24;3 years), including 24 male and 15 female participants. The absence of language impairment or delay was confirmed through formal language tests and questionnaires (see [7]). A detailed description of the inclusion and exclusion criteria of participants can be found in Svaldi et al. [7] and patient characteristics are reported in the Supplementary materials (reprint from Svaldi et al. [7]). See Appendix A for the cognitive status of patients within one year before language assessment.

### Procedure

Spontaneous language samples (minimum 300 words) were gathered based on structured conversations and picture descriptions and were transcribed following the methods outlined in Svaldi et al. [7]. Patient data were collected in 2007 in one testing session. Control data were collected in 2020 and 2021 and consisted of two testing sessions since additional language tests were administered in the first session to rule out language impairment.

### Data Coding and Analysis

The samples analyzed by Svaldi et al. [7] were coded for the types of (lexico-) phonological, lexical-semantic and morphosyntactic errors produced (see Appendix B for descriptions of the error types). The proportion of each error type was calculated by dividing the number of errors in the whole sample (i.e., conversation + picture descriptions) by the total number of utterances. The error proportions were compared between each patient and their individually matched set of five controls using modified *t*-tests for single-case statistics [10].

## Results

Each patient produced a greater proportion of errors than controls on at least one of the error types. Significant differences occurred across all language processing levels and are indicative of impaired spontaneous language (see Table 1). Findings of increased error occurrence in patients appeared to be more frequent in the pCMS-group (19 significant findings in five patients) than the non-pCMS-group (12 significant findings in seven patients). Descriptive statistics of the proportions of errors produced and results of the individual case statistics can be found in the Supplementary materials. The level of significance was set at α < 0.05.

**Table 1.**
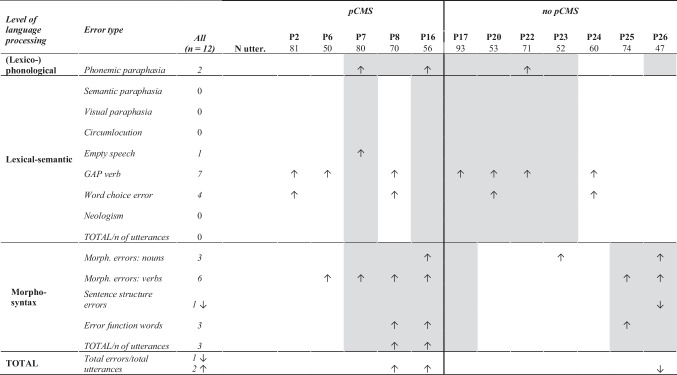
Results of individual comparisons of error proportions between cerebellar tumor survivors and controls. Arrows indicate a significantly higher (↑) or lower (↓) proportion of an error type compared to controls. Grey shading indicates language processing levels for which there was evidence of impairment in Svaldi et al. [7]

*All* = Total number of patients that scored significantly higher/lower than controls; *pCMS* = Postoperative cerebellar mutism syndrome; *N utter*. = Number of utterances produced by the patient; *GAP* = General-all-purpose; *Morph*. = Morphological

Three out of 12 patients, of whom two were diagnosed with pCMS, produced (lexico-) phonological errors (i.e., phonemic paraphasias). Lexical-semantic errors were observed in eight patients. The production of general-all-purpose (GAP) verbs was the most common lexical-semantic error for individuals with and without pCMS. Seven out of 12 patients produced morphosyntactic errors and, while keeping in mind small numbers, tended to occur more in the pCMS-group (4/5 in pCMS-group relative to 3/7 in non-pCMS-group). Here, morphological errors in verb inflection were most often observed, particularly in the pCMS-group.

For eight patients, error patterns aligned with the observations of Svaldi et al. [7] based on standard spontaneous language measures and psycholinguistic properties (see Table 1). For three patients (P23, P17, P16) the psycholinguistic analysis detected impairments in lexical-semantics or morphosyntax that were not confirmed by the error analysis. A greater mismatch was observed for (lexico-)phonological processing, where the error analyses did not reveal significant findings in five out of eight patients in whom the psycholinguistic analysis indicated a phonological impairment.

In contrast, four other patients (P2, P6, P8, P24) showed increased lexical-semantic errors, despite no difference to controls in spontaneous language measures included in Svaldi et al. [7]. This was possibly due to limited measurable spontaneous language for two of these patients (P2 and P6), which led to exclusion from some comparisons. For two patients (P6, P23), error analyses revealed additional morphosyntactic difficulties.

## Discussion and Conclusion

The findings of this error analysis demonstrated that long-term (lexico-)phonological, lexical-semantic and morphosyntactic impairments may be present in cerebellar tumor survivors, in the presence of normal scores in other cognitive domains. This aligns with earlier studies and supplements previous reports with a more detailed evaluation of language processing abilities [5, 7, 11]. Such comprehensive evaluation seems thus warranted and necessary for targeted language therapy [2].

Lexical-semantic and morphosyntactic errors were most common in our patient group, aligning with earlier reports on difficulties in these language levels [5, 11, 12]. An increased number of GAP verbs was the most common lexical-semantic error and may reflect immature language or word-finding difficulties [13]. Verb inflection mistakes were the most common morphosyntactic errors and have previously been reported in this population [5]. Further research is necessary, however, to confirm these findings which should include more phonological error types (e.g., phonemic distortions, differentiating between programming and selection impairment).

More differences between patients and controls were found in the pCMS-group compared to the non-pCMS-group. Morphosyntactic disorders may be particularly frequent in the pCMS-group [6]. Notably, preoperative morphosyntactic disorders were suggested as risk factors for pCMS [11]. This could mean that the higher proportion of morphosyntactic errors in the pCMS-group reflects pre-surgical impairments. However, this can only be established in future research including a preoperative assessment and equal groups of patients with and without pCMS, as well as a larger sample size so that more generalizable patterns may emerge. Similarly, prevalence data is notoriously difficult to establish in small-N studies.

It should also be considered that the occurrence of pCMS may result in less reliable spontaneous language assessment, given the reduced output. However, where standard and psycholinguistic measures detected no impairments for some patients [7], the error analysis did. The opposite was also observed, indicating that different analyses can provide complementary information, potentially tapping into different underlying impairments. A combination of standard measures, psycholinguistic analyses, and error analyses may increase the sensitivity to identify impairments and to characterize their language processing nature [13].

The error analysis conducted in the present study suggested long-term language impairment in cerebellar tumor survivors. A detailed and individualized language follow-up seems necessary to investigate the processing nature of these impairments.

## Supplementary Material

Below is the link to the electronic supplementary material.

Supplementary file1 (DOCX 43.1 KB)

## Data Availability

The patient data analyzed during the current study were shared with us and requests for data sharing should go to Prof Philippe Paquier. Control data are available from the corresponding author on reasonable request and can be shared via DataverseNL.

## Appendix A

See Table 2

**Table 2:** Cognitive status of patients within one year before spontaneous language assessment based on retrospective evaluation of clinical records

Case	Age at language assessment (yy;mm)	Age at cognitive assessment (yy;mm)	School information		IQ				Memory		
			Group	School type	Test	Total	Verbal	Performal	Verbal memory		Non-verbal long-term memory
									Short-term	Long-term	
*P6*	6;0	5;5	2 (age-appropriate)	Regular	WPPSI-R	94 (average)	100 (average)	87 (average)	-	-	-
*P8*	7;8	No cognitive assessment									
*P2*	8;10	8;4	4 (age-appropriate)	Regular	WISC-III	76 (below average)	82 (average)	73 (below average)	Low average	Below average	Below average
*P7*	19;9	19;3	NA	NA	GIT-2	93 (average)	-	-	Below average	Below average	Average
*P16*	24;2	No cognitive assessment									
*P17*	3;0	3;1	NA	NA	SON-R	113 (average)	110 (average)	114 (average)	-	-	
*P25*	6;7	5;7	3 (1 year behind)	Regular	WISC-III	68 (below average)	70 (below average)	72 (below average)	Average	Average	Far below average
*P24*	8;1	No cognitive assessment									
*P20*	10;2	9;8	5 (1 year behind)	Regular	WISC-III	87 (average)	80 (average)	99 (average)	Above average	Average	Below average
*P23*	11;1	No cognitive assessment									
*P26*	11;5	11	7 (age-appropriate)	Regular	WISC-III	109 (average)	107 (average)	109 (average)	Average	Average	Average
*P22*	18;3	-	No information	No information	WAIS-III	-	93 (average)	-	-	-	-

Case	Age at language assessment (yy;mm)	Age at cognitive assessment (yy;mm)	Sustained attention		Selective attention	Visuo-spatial skills	Executive functioning	
			Speed	Accuracy			Part A (speed/accuracy)	Part B (speed/accuracy)
*P6*	6;0	5;5	-	-	-	-	-	-
*P8*	7;8	No cognitive assessment						
*P2*	8;10	8;4	-	-	-	Below average	-	-
*P7*	19;9	19;3	Far below average	Above average	-	Average	Far below average/Average	Low average
*P16*	24;2	No cognitive assessment						
*P17*	3;0	3;1	-	-	-	-	-	-
*P25*	6;7	5;7	Far below average	-	-	Far below average	Far below average/Average	-
*P24*	8;1	No cognitive assessment						
*P20*	10;2	9;8	Average	Above average	Far below average	Average	Average	Below average/Average
*P23*	11;1	No cognitive assessment						
*P26*	11;5	11	Average	Above average	Above average	Average	Far below average/Average	Average
*P22*	18;3	-	-	-	-	-	-	-

*Group* = Dutch primary school group ranging from 1 (4–5 years) to 8 (11–12 years); *IQ* = Intelligence Quotient; *WPPSI-R* = Wechsler Preschool and Primary Scale of Intelligence Revised; *WISC-III* = Third version of the Wechsler Intelligence Scale for Children; *GIT-2* = Groningen Intelligence Test 2; *SON-R* = Snijders-Oomen Non-Verbal Intelligence Test; *WAIS-III* = Third version of the Wechsler Adult Intelligence Scale; Verbal memory = 15-Word Test; *Non-verbal long-term memory* = Rey Complex Figure Test; *Sustained attention* = Bourdon-Vos Test;* Selective attention* = Stroop task; *Visuo-spatial skills* = Rey Complex Figure Test; *Executive functioning* = Trail Making Test.

## Appendix B

See Table 3

**Table 3:** Description of the error types in spontaneous language per level of language processing

Level of language processing	Error type	Description	Example
(Lexico-)phonological	*Phonemic paraphasia*	Substitution, addition, transposition or deletion of one or more phonemes of the target word	‘fidge’ instead of ‘f*r*idge’
Lexical-semantic	*Semantic paraphasia*	The target word is replaced with a word with a similar meaning	‘table’ instead of ‘chair’
	*Visual paraphasia*	The target word is replaced with a word that is visually similar	‘stick’ instead of ‘pipe’
	*Circumlocution*	A description of the target word or concept is given instead of producing it	‘the father of my father’ instead of ‘grandfather’
	*Empty speech*	Utterance characterized by general, vague, unspecific referents, but is semantically and syntactically correct	‘*that thing* is blue’ instead of ‘*the sky* is blue’
	*General-all-purpose verbs*	A general non-specific verb is produced instead of a specific verb to describe an action	‘I *did* school’ instead of ‘I *attended* school’
	*Word choice errors*	A lexically incorrect word is chosen, making an utterance incorrect within the context of the other words within that utterance or the sample	‘I like to *eat* juice’ instead of ‘I like to *drink* juice’
	*Neologism*	A nonword substitution of the target word with less than 50% overlap in phonemes with the target word	‘bronny’ instead of ‘sweater’
Morphosyntax	*Morphological errors* ^*a*^	An error in the inflection or conjugation of a noun or verb	‘child*s*’ instead of ‘children’
	*Sentence structure errors*	Incorrect syntactic structure of an utterance caused by incorrect word order, an incomplete sentence or deletion of a part of the structure (e.g., a finite verb, subject, object)	‘walk the dog everyday’ instead of ‘*I* walk the dog everyday’
	*Errors in usage of function words*	Substitution, deletion or addition of a preposition, pronoun, article, adverb or conjunction that is incorrect in the context of the utterance	‘He was sitting *in* the bench’ instead of ‘He was sitting *on* the bench’

^*a*^ = Separate for nouns and verbs
