# Supplementary material for: Errors in the Spontaneous Language of Survivors of Pediatric Cerebellar Tumors
Source: Cerebellum. 2025 Jan 7;24(1):26. doi: 10.1007/s12311-024-01754-2 (PMC11703980; doi:10.1007/s12311-024-01754-2)
Supplement: Supplementary file 1 — Supplementary file1 (DOCX 43.1 KB) [file 12311_2024_1754_MOESM1_ESM.docx]

**Supplementary Materials**

**Table 1** Demographic, tumour, and tumour treatment characteristics of the participants who underwent cerebellar tumour surgery (n = 12)

| **Group** | **Case** | **Gender** | **Age at assessment (yy;mm)** | **Time since surgery (yy;mm)** | **Duration mutism (days)** | **Tumour type** | **Tumour diameter (cm)** | **Tumour location** | **Hydrocephalus (BI)** | | **Treatment** | **Extent of resection** | **Age controls (*M* (*SD*)) (yy;mm)** |
| --- | --- | --- | --- | --- | --- | --- | --- | --- | --- | --- | --- | --- | --- |
|  |  |  |  |  |  |  |  |  | **Pre-op** | **Post-op** |  |  |  |
| *pCMS* | *P6* | M | 6;0 | 2;10 | 29 | PA | 3.5 | 4th V + pons + RCH | 0.26* | 0.10 | S + C | Subtotal | 6;4 (0;3) |
|  | *P8* | F | 7;8 | 2;1 | 51 | PA | 5 | Vermis + 4th V | 0.27* | 0.03 | S | Total | 7;10 (0;4) |
|  | *P2* | F | 8;10 | 4;2 | 21 | MB | 4.8 | Vermis | 0.24* | 0.19 | S + C + R | Subtotal | 7;10 (0;4) |
|  | *P7* | M | 19;9 | 2 | 70 | MB | 4.3 | Vermis | 0.25* | 0.11 | S + C + R | Total | 21;6 (1;11) |
|  | *P16* | M | 24;2 | 12;3 | 152 | MB | 4.1 | Vermis | 0.19 | 0.14 | S + C + R | Total | 21;6 (1;11) |
| *no pCMS* | *P17* | M | 3;0 | 0;11 | N.A. | EP | 3.5 | Vermis + LCH | 0.20* | 0.10 | S + R | Subtotal | 3;4 (0;3) |
|  | *P25* | M | 6;7 | 4;2 | N.A. | MB | 5 | Vermis | 0.15 | 0.14 | S + C + R | Subtotal | 6;4 (0;3) |
|  | *P24* | M | 8;1 | 1;2 | N.A. | MB | 4.6 | Vermis | 0.28* | 0.15 | S + C + R | Subtotal | 9 (0;4) |
|  | *P20* | M | 10;2 | 2;4 | N.A. | EP | 2.3 | Vermis | 0.10 | 0.14 | S + R | Total | 9;11 (0;4) |
|  | *P23* | F | 11;1 | 1;10 | N.A. | PA | 6.3 | Vermis + LCH | 0.28* | 0.13 | S | Total | 11;10 (0;4) |
|  | *P26* | F | 11;5 | 7;4 | N.A. | PA | 4.2 | Vermis | 0.43* | 0.05 | S | Total | 11;10 (0;4) |
|  | *P22* | F | 18;3 | 8 | N.A. | PA | 4 | Vermis + 4th V + LCH | 0.37* | 0.1 | S | Subtotal | 18;5 (0;5) |

*pCMS* = postoperative cerebellar mutism syndrome; *P* = patient; *yy;mm* = years;months; *N.A.* = not applicable; *PA* = pilocytic astrocytoma; *MB* = medulloblastoma; *EP* = ependymoma; *4^th^ V* = fourth ventricle; *RCH* = right cerebellar hemisphere; *LCH* = left cerebellar hemisphere; *BI* = bicaudate index; * = hydrocephalus; *S* = surgery; *C* = chemotherapy; *R* = radiotherapy

**Table 2** Individual scores for each error type for the pCMS-group (n = 5) and their controls.

| **Level of language processing** | **Error type** | **Error proportion** | | | | **Error proportion** | | | |
| --- | --- | --- | --- | --- | --- | --- | --- | --- | --- |
|  |  | P2 | Controls  *M*(*SD*) | *p*-value | *T*-value | P6 | Controls  *M*(*SD*) | *p*-value | *T*-value |
| ***(Lexico-) phonological*** | *Phonemic paraphasia* | 0.07 | 0.03(0.03) | .126 | 1.34 | 0.04 | 0.02(0.02) | .219 | 0.86 |
| ***Lexical-semantic*** | *Semantic paraphasia* | NA | NA | .336 | -0.46 | NA | NA | .358 | -0.39 |
|  | *Visual paraphasia* | 0.01 | 0.00(0.00) | NA | - | 0.02 | 0.00(0.00) | NA | - |
|  | *Circumlocution* | 0.00 | 0.01(0.01) | .231 | -0.81 | NA | NA | NA | - |
|  | *Empty speech* | 0.10 | 0.15(0.10) | .319 | -0.51 | 0.10 | 0.10(0.05) | .487 | -0.04 |
|  | *GAP verb* | 0.14 | 0.04(0.02) | **.008*** | **3.97** | 0.04 | 0.00(0.01) | **.004*** | **4.83** |
|  | *Word choice error* | 0.12 | 0.03(0.02) | **.003*** | **5.42** | 0.06 | 0.03(0.02) | .159 | 1.14 |
|  | *Neologism* | NA | NA | .356 | -0.46 | NA | NA | NA | - |
|  | *TOTAL/n of utterances* | 0.37 | 0.23(0.12) | .174 | 1.06 | 0.22 | 0.14(0.07) | .163 | 1.12 |
| ***Morphosyntax*** | *Morphological errors: nouns* | 0.00 | 0.01(0.02) | .353 | -0.41 | 0.00 | 0.01(0.01) | .188 | -1.00 |
|  | *Morphological errors: verbs* | 0.04 | 0.02(0.02) | .301 | 0.57 | 0.04 | 0.02(0.01) | **.032*** | **2.54** |
|  | *Error sentence structure* | 0.07 | 0.11(0.03) | .219 | -0.86 | 0.10 | 0.08(0.05) | .398 | 0.28 |
|  | *Error function words* | 0.11 | 0.09(0.01) | .104 | 1.50 | 0.12 | 0.13(0.04) | .402 | -0.26 |
|  | *TOTAL/n of utterances* | 0.22 | 0.23(0.05) | .471 | -0.08 | 0.26 | 0.24(0.07) | .418 | 0.22 |

**Note.** *pCMS* = Postoperative cerebellar mutism syndrome; *GAP* = General-all-purpose; *** = *p* < .05.

**Table 2** (continued)

| **Level of language processing** | **Error type** | **Error proportion** | | | | **Error proportion** | | | |
| --- | --- | --- | --- | --- | --- | --- | --- | --- | --- |
|  |  | P7 | Controls  *M*(*SD*) | *p*-value | *T*-value | P8 | Controls  *M*(*SD*) | *p*-value | *T*-value |
| ***(Lexico-) phonological*** | *Phonemic paraphasia* | 0.10 | 0.02(0.03) | **.041*** | **2.31** | 0.06 | 0.03(0.03) | .229 | 0.82 |
| ***Lexical-semantic*** | *Semantic paraphasia* | NA | NA | NA | - | NA | NA | NA | - |
|  | *Visual paraphasia* | NA | NA | NA | - | NA | NA | NA | - |
|  | *Circumlocution* | 0.00 | 0.01(0.02) | .346 | -0.43 | 0.01 | 0.01(0.01) | .288 | 0.61 |
|  | *Empty speech* | 0.18 | 0.09(0.03) | **.028*** | **2.68** | 0.10 | 0.15(0.10) | .322 | -0.50 |
|  | *GAP verb* | 0.03 | 0.03(0.03) | .432 | -0.18 | 0.10 | 0.04(0.02) | **.032*** | **2.54** |
|  | *Word choice error* | 0.04 | 0.04(0.03) | .469 | 0.08 | 0.07 | 0.03(0.02) | **.044*** | **2.25** |
|  | *Neologism* | NA | NA | NA | - | NA | NA | NA | - |
|  | *TOTAL/n of utterances* | 0.24 | 0.16(0.09) | .242 | 0.77 | 0.33 | 0.23(0.12) | .250 | 0.74 |
| ***Morphosyntax*** | *Morphological errors: nouns* | 0.01 | 0.01(0.01) | .476 | 0.07 | 0.00 | 0.01(0.02) | .353 | -0.41 |
|  | *Morphological errors: verbs* | 0.05 | 0.01(0.01) | **.002*** | **5.87** | 0.14 | 0.02(0.02) | **.003*** | **5.17** |
|  | *Error sentence structure* | 0.15 | 0.16(0.04) | .398 | -0.28 | 0.13 | 0.11(0.03) | .285 | 0.62 |
|  | *Error function words* | 0.08 | 0.11(0.04) | .250 | -0.74 | 0.27 | 0.09(0.01) | **<.001*** | **11.93** |
|  | *TOTAL/n of utterances* | 0.29 | 0.29(0.02) | .481 | -0.05 | 0.54 | 0.23(0.05) | **.002*** | **6.16** |

**Note.** *GAP* = General-all-purpose; *** = *p* < .05.

**Table 2** (continued)

| **Level of language processing** | **Error type** | **Error proportion** | | | |
| --- | --- | --- | --- | --- | --- |
|  |  | P16 | Controls  *M*(*SD*) | *p*-value | *T*-value |
| ***(Lexico-) phonological*** | *Phonemic paraphasia* | 0.14 | 0.02(0.03) | **.011*** | **3.62** |
| ***Lexical-semantic*** | *Semantic paraphasia* | NA | NA | NA | - |
|  | *Visual paraphasia* | NA | NA | NA | - |
|  | *Circumlocution* | 0.00 | 0.01(0.02) | .346 | -0.43 |
|  | *Empty speech* | 0.11 | 0.09(0.03) | .288 | 0.61 |
|  | *GAP verb* | 0.00 | 0.03(0.03) | .199 | -0.94 |
|  | *Word choice error* | 0.05 | 0.04(0.03) | .313 | 0.53 |
|  | *Neologism* | NA | NA | NA | - |
|  | *TOTAL/n of utterances* | 0.16 | 0.16(0.09) | .492 | -0.02 |
| ***Morphosyntax*** | *Morphological errors: nouns* | 0.05 | 0.01(0.01) | **.026*** | **2.74** |
|  | *Morphological errors: verbs* | 0.05 | 0.01(0.01) | **.002*** | **6.39** |
|  | *Error sentence structure* | 0.18 | 0.16(0.04) | .376 | 0.34 |
|  | *Error function words* | 0.25 | 0.11(0.04) | **.019*** | **3.07** |
|  | *TOTAL/n of utterances* | 0.54 | 0.29(0.02) | **<.001*** | **12.53** |

**Note.** *GAP* = General-all-purpose; *** = *p* < .05.

**Table 3** Individual scores for each error type for the non-pCMS-group (n = 7) and their controls.

| **Level of language processing** | **Error type** | **Error proportion** | | | | **Error proportion** | | | |
| --- | --- | --- | --- | --- | --- | --- | --- | --- | --- |
|  |  | P17 | Controls  *M*(*SD*) | *p*-value | *T*-value | P20 | Controls  *M*(*SD*) | *p*-value | *T*-value |
| ***(Lexico-) phonological*** | *Phonemic paraphasia* | 0.08 | 0.23(0.31) | .334 | -0.46 | 0.04 | 0.02(0.02) | .196 | 0.96 |
| ***Lexical-semantic*** | *Semantic paraphasia* | 0.00 | 0.01(0.01) | .336 | -0.46 | 0.04 | 0.00(0.01) | **.013*** | **3.45** |
|  | *Visual paraphasia* | NA | NA | NA | - | NA | NA | NA | - |
|  | *Circumlocution* | 0.00 | 0.01(0.01) | .307 | -0.55 | NA | NA | NA | - |
|  | *Empty speech* | 0.13 | 0.12(0.09) | .477 | 0.06 | 0.06 | 0.16(0.14) | .272 | -0.66 |
|  | *GAP verb* | 0.03 | 0.00(0.01) | **.010*** | **3.78** | 0.09 | 0.02(0.02) | **.006*** | **4.44** |
|  | *Word choice error* | 0.04 | 0.02(0.01) | .112 | 1.44 | 0.11 | 0.03(0.02) | **.011*** | **3.61** |
|  | *Neologism* | 0.02 | 0.01(0.01) | .114 | 1.42 | 0.00 | 0.01(0.02) | .346 | -0.43 |
|  | *TOTAL/n of utterances* | 0.25 | 0.17(0.08) | .209 | 0.90 | 0.30 | 0.22(0.15) | .326 | 0.49 |
| ***Morphosyntax*** | *Morphological errors: nouns* | NA | NA | NA | - | NA | NA | NA | - |
|  | *Morphological errors: verbs* | 0.00 | 0.07(0.04) | .110 | -1.46 | 0.02 | 0.01(0.01) | .167 | 1.10 |
|  | *Error sentence structure* | 0.23 | 0.13(0.05) | .077 | 1.76 | 0.13 | 0.12(0.05) | .440 | 0.16 |
|  | *Error function words* | 0.15 | 0.15(0.05) | .493 | -0.02 | 0.13 | 0.11(0.05) | .352 | 0.41 |
|  | *TOTAL/n of utterances* | 0.38 | 0.35(0.14) | .433 | 0.18 | 0.28 | 0.24(0.08) | .318 | 0.51 |

**Note.** *pCMS* = Postoperative cerebellar mutism syndrome; *GAP* = General-all-purpose; *** = *p* < .05.

**Table 3** (Continued)

| **Level of language processing** | **Error type** | **Error proportion** | | | | **Error proportion** | | | |
| --- | --- | --- | --- | --- | --- | --- | --- | --- | --- |
|  |  | P22 | Controls  *M*(*SD*) | *p*-value | *T*-value | P23 | Controls  *M*(*SD*) | *p*-value | *T*-value |
| ***(Lexico-) phonological*** | *Phonemic paraphasia* | 0.06 | 0.01(0.01) | **.003*** | **5.14** | 0.02 | 0.03(0.04) | .382 | -0.32 |
| ***Lexical-semantic*** | *Semantic paraphasia* | NA | NA | NA | - | 0.00 | 0.01(0.01) | .288 | -0.61 |
|  | *Visual paraphasia* | NA | NA | NA | - | NA | NA | NA | - |
|  | *Circumlocution* | NA | NA | NA | - | 0.00 | 0.01(0.01) | .288 | -0.61 |
|  | *Empty speech* | 0.00 | 0.08(0.05) | .094 | -1.59 | 0.06 | 0.23(0.10) | .096 | -1.57 |
|  | *GAP verb* | 0.03 | 0.01(0.01) | **.033*** | **2.51** | 0.04 | 0.03(0.03) | .365 | 0.37 |
|  | *Word choice error* | 0.03 | 0.01(0.01) | .178 | 1.04 | 0.06 | 0.03(0.03) | .199 | 0.94 |
|  | *Neologism* | NA | NA | NA | - | NA | NA | NA | - |
|  | *TOTAL/n of utterances* | 0.06 | 0.10(0.04) | .192 | -0.98 | 0.15 | 0.29(0.08) | .095 | -1.58 |
| ***Morphosyntax*** | *Morphological errors: nouns* | NA | NA | NA | - | 0.02 | 0.00(0.01) | **.036*** | **2.43** |
|  | *Morphological errors: verbs* | 0.00 | 0.01(0.01) | .281 | -0.63 | 0.02 | 0.01(0.01) | .155 | 1.16 |
|  | *Error sentence structure* | 0.17 | 0.13(0.08) | .344 | 0.43 | 0.19 | 0.15(0.03) | .117 | 1.40 |
|  | *Error function words* | 0.03 | 0.09(0.05) | .152 | -1.18 | 0.12 | 0.08(0.05) | .279 | 0.64 |
|  | *TOTAL/n of utterances* | 0.20 | 0.23(0.07) | .342 | -0.44 | 0.35 | 0.23(0.07) | .095 | 1.58 |

**Note.** *pCMS* = Postoperative cerebellar mutism syndrome; *GAP* = General-all-purpose; *** = *p* < .05.

**Table 3** (Continued)

| **Level of language processing** | **Error type** | **Error proportion** | | | | **Error proportion** | | | |
| --- | --- | --- | --- | --- | --- | --- | --- | --- | --- |
|  |  | P24 | Controls  *M*(*SD*) | *p*-value | *T*-value | P25 | Controls  *M*(*SD*) | *p*-value | *T*-value |
| ***(Lexico-) phonological*** | *Phonemic paraphasia* | 0.00 | 0.02(0.02) | .248 | -0.75 | 0.04 | 0.02(0.02) | .206 | 0.91 |
| ***Lexical-semantic*** | *Semantic paraphasia* | NA | NA | NA | - | NA | NA | NA | - |
|  | *Visual paraphasia* | NA | NA | NA | - | NA | NA | NA | - |
|  | *Circumlocution* | NA | NA | NA | - | NA | NA | NA | - |
|  | *Empty speech* | 0.08 | 0.13(0.07) | .282 | -0.63 | 0.12 | 0.10(0.05) | .372 | 0.35 |
|  | *GAP verb* | 0.08 | 0.02(0.02) | **.007*** | **4.14** | NA | NA | NA | - |
|  | *Word choice error* | 0.12 | 0.04(0.03) | **.025*** | **2.78** | 0.04 | 0.03(0.02) | .349 | 0.42 |
|  | *Neologism* | 0.00 | 0.01(0.01) | .266 | -0.69 | NA | NA | NA | - |
|  | *TOTAL/n of utterances* | 0.28 | 0.20(0.09) | .226 | 0.83 | 0.20 | 0.14(0.07) | .213 | 0.89 |
| ***Morphosyntax*** | *Morphological errors: nouns* | NA | NA | NA | - | 0.01 | 0.01(0.01) | .438 | 0.17 |
|  | *Morphological errors: verbs* | 0.00 | 0.01(0.01) | .294 | -0.59 | 0.05 | 0.02(0.01) | **.008*** | **3.96** |
|  | *Error sentence structure* | 0.18 | 0.12(0.08) | .242 | 0.77 | 0.05 | 0.08(0.05) | .316 | -0.52 |
|  | *Error function words* | 0.05 | 0.09(0.04) | .222 | -0.85 | 0.04 | 0.13(0.04) | **.049*** | **-2.16** |
|  | *TOTAL/n of utterances* | 0.23 | 0.22(0.11) | .460 | 0.11 | 0.16 | 0.24(0.07) | .190 | -0.99 |

**Note.** *pCMS* = Postoperative cerebellar mutism syndrome; *GAP* = General-all-purpose; *** = *p* < .05.

**Table 3** (continued)

| **Level of language processing** | **Error type** | **Error proportion** | | | |
| --- | --- | --- | --- | --- | --- |
|  |  | P26 | Controls  *M*(*SD*) | *p*-value | *T*-value |
| ***(Lexico-) phonological*** | *Phonemic paraphasia* | 0.06 | 0.03(0.04) | .237 | 0.79 |
| ***Lexical-semantic*** | *Semantic paraphasia* | 0.00 | 0.01(0.01) | .288 | -0.61 |
|  | *Visual paraphasia* | NA | NA | NA | - |
|  | *Circumlocution* | 0.00 | 0.01(0.01) | .288 | -0.61 |
|  | *Empty speech* | 0.02 | 0.23(0.10) | .064 | -1.92 |
|  | *GAP verb* | 0.04 | 0.03(0.03) | .317 | 0.51 |
|  | *Word choice error* | 0.04 | 0.03(0.03) | .331 | 0.47 |
|  | *Neologism* | NA | NA | NA | - |
|  | *TOTAL/n of utterances* | 0.11 | 0.29(0.08) | .051 | -2.12 |
| ***Morphosyntax*** | *Morphological errors: nouns* | 0.02 | 0.00(0.01) | **.026*** | **2.74** |
|  | *Morphological errors: verbs* | 0.04 | 0.01(0.01) | **.017*** | **3.15** |
|  | *Error sentence structure* | 0.06 | 0.15(0.03) | **.034*** | **-2.50** |
|  | *Error function words* | 0.04 | 0.08(0.05) | .290 | -0.60 |
|  | *TOTAL/n of utterances* | 0.17 | 0.23(0.07) | .220 | -0.86 |

**Note.** *GAP* = General-all-purpose; *** = *p* < .05.
